# Antibodies against Spike protein correlate with broad autoantigen recognition 8 months post SARS-CoV-2 exposure, and anti-calprotectin autoantibodies associated with better clinical outcomes

**DOI:** 10.3389/fimmu.2022.945021

**Published:** 2022-08-11

**Authors:** Rhiane Moody, Sabrina Sonda, Fay H. Johnston, Kylie J. Smith, Nicola Stephens, Michelle McPherson, Katie L. Flanagan, Magdalena Plebanski

**Affiliations:** ^1^ School of Health and Biomedical Science, STEM College, RMIT University, Bundoora, VIC, Australia; ^2^ Tasmanian Vaccine Trial Centre, Clifford Craig Foundation, Launceston General Hospital, Launceston, TAS, Australia; ^3^ School of Health Sciences and School of Medicine, University of Tasmania, Launceston, TAS, Australia; ^4^ Public Health Services, Department of Health, Tasmania, TAS, Australia; ^5^ Menzies Institute for Medical Research, University of Tasmania, Hobart, TAS, Australia; ^6^ Tasmanian School of Medicine, University of Tasmania, Hobart, TAS, Australia

**Keywords:** SARS-CoV-2, COVID-19, antibodies, autoimmunity, autoantibodies

## Abstract

Autoantibodies to multiple targets are found during acute COVID-19. Whether all, or some, persist after 6 months, and their correlation with sustained anti-SARS-CoV-2 immunity, is still controversial. Herein, we measured antibodies to multiple SARS-CoV-2 antigens (Wuhan-Hu-1 nucleoprotein (NP), whole spike (S), spike subunits (S1, S2 and receptor binding domain (RBD)) and Omicron spike) and 102 human proteins with known autoimmune associations, in plasma from healthcare workers 8 months post-exposure to SARS-CoV-2 (n=31 with confirmed COVID-19 disease and n=21 uninfected controls (PCR and anti-SARS-CoV-2 negative) at baseline). IgG antibody responses to SARS-CoV-2 antigens were significantly higher in the convalescent cohort than the healthy cohort, highlighting lasting antibody responses up to 8 months post-infection. These were also shown to be cross-reactive to the Omicron variant spike protein at a similar level to lasting anti-RBD antibodies (correlation r=0.89). Individuals post COVID-19 infection recognised a common set of autoantigens, specific to this group in comparison to the healthy controls. Moreover, the long-term level of anti-Spike IgG was associated with the breadth of autoreactivity post-COVID-19. There were further moderate positive correlations between anti-SARS-CoV-2 responses and 11 specific autoantigens. The most commonly recognised autoantigens were found in the COVID-19 convalescent cohort. Although there was no overall correlation in self-reported symptom severity and anti-SARS-CoV-2 antibody levels, anti-calprotectin antibodies were associated with return to healthy normal life 8 months post infection. Calprotectin was also the most common target for autoantibodies, recognized by 22.6% of the overall convalescent cohort. Future studies may address whether, counter-intuitively, such autoantibodies may play a protective role in the pathology of long-COVID-19.

## 1 Introduction

Coronavirus Disease-19 (COVID-19), caused by severe acute respiratory syndrome coronavirus-2 (SARS-CoV-2) can result in a range of clinical outcomes and manifestations (1–3). Although rare, an aspect of these manifestations includes various autoimmune and autoimmune-like diseases, such as Guillain-Barré syndrome, multisystem inflammatory syndrome and systemic lupus erythematosus (SLE) [reviewed in (4)]. In addition to these autoimmune-like diseases, there is a growing number of people with long-COVID or post-COVID-19 syndrome, which emerging studies suggest have immune and autoimmune factors in aetiology (5–8).

Antibody responses to the Spike, S1, RBD and Nucleoprotein of SARS-CoV-2 have been shown to last up to a year post disease (9–12). In the initial months post infection, studies report the antibody responses to multiple SARS-CoV-2 antigens (13, 14), however, less is known about the pattern of persistence to multiple antigens, within the same individuals, post 6 months. During the acute phase of infection, the level of antibody responses has been reported to be associated with disease severity, where those with more severe disease had greater antibody levels (13, 15). Contradictory evidence exists on their persistence, with this association reported by some (16–18), but not all (19, 20) studies to continue post-disease. Additionally, reports on the level of the long-term anti-SARS-CoV-2 responses and associations to clinical outcomes are few, although current studies suggest they have no impact on long-COVID (8, 16, 21). With multiple variants emerging since the beginning of the pandemic, understanding cross-reactive immune responses based on prior infection is important for understanding protection against newer variants. Prior infection with Wuhan variant can result in cross-reactive antibodies to the Beta, Delta and Gamma variants (22), and neutralising antibody responses of different variants occur based on shared spike mutations (23). Of latest concern is the Omicron variant and subvariants. The initial Omicron variant (B.1.1.529) was found to escape neutralisation from pre-exposed serum, as well as recipients of 2 mRNA vaccine doses, and instead required a third vaccine dose (23). However, antibodies to the Omicron spike protein have been reported in response to vaccinations (24). To our knowledge, persistence of cross-reactive antibodies, irrespective of neutralisation, between an earlier Wuhan infection and Omicron, without intervening vaccination, has not been investigated.

The presence of autoantibodies to various autoantigens has been described in COVID-19 patients (25–27). There are reports of severe COVID-19 cases with autoantibodies to SSA/Ro (25), cardiolipin (26), beta 1 glycoprotein I (β1GP1) (26) as well as positive antinuclear antibodies (ANAs) (25, 26). Bastard et al. additionally reported autoantibodies to type I interferons (IFNs) in COVID-19 patients, which were associated with acute severe but not mild disease (27). Protein microarrays have additionally been used to identify autoantibody responses on a larger scale, to antigens both with and without known autoimmune associations (28–30). Emerging studies have assessed large-scale autoantibody responses up to six (29) or eight (5) months post-infection. However, to our knowledge, there are no studies comparing the broad range of autoantibodies present in healthy individuals compared to individuals with persistent sequelae following a single natural challenge event; or the association of reactivity or autoreactivity to multiple SARS-CoV-2 antigens, 8 months post exposure with different clinical outcomes.

In the present study, microarray chips consisting of 102 known autoantigens and 6 SARS-CoV-2 antigens were used to measure the IgG antibody responses in plasma samples from COVID-19 convalescent individuals and uninfected controls. Collected 8 months after a single exposure event with the original Wuhan-Hu-1 variant, we sought to further explore lasting anti-SARS-CoV-2 antibody responses and the potential long-term association of autoreactive immunity with COVID-19 responses.

## 2 Materials and methods

### 2.1 Clinical cohort

Between 20^th^ March and 13^th^ April 2020, a COVID-19 outbreak occurred at two co-located hospitals and associated health care services in Tasmania, Australia (31). By the end of the outbreak there were 138 cases including 80 healthcare workers. Healthcare workers (n=1,779), including clinical and non-clinical staff, who worked at the two hospitals during the outbreak were invited to complete an online survey. The survey collected a variety of information including demographic details, if they had COVID-19, and symptoms experienced during the outbreak period of 20^th^ March to 10^th^ May 2020 which includes the 14 days after completion of the compulsory quarantine period.

During December 2020, approximately eight months after the outbreak, 88 participants attended a study clinic, where they gave a blood sample and completed another questionnaire, again reporting on symptoms experienced during 20^th^ March to 10^th^ May 2020, the severity of their symptoms during the outbreak and if they were feeling back to normal. The symptoms data from this survey was used only if the symptom question was not answered in the earlier online survey. Plasma from the whole blood samples collected was isolated using standardised Ficoll-gradient separation and stored at -80 degrees Celsius prior to use. Following the control of this outbreak, no community transmission of COVID-19 occurred in Tasmania until December 2021 (32). Based on PCR data available, the present study used plasma from 31 PCR confirmed positives and corresponding 30 PCR confirmed negatives for SARS-CoV-2 infection.

This study was conducted according to the guidelines of the Declaration of Helsinki and approved by Tasmanian Health and Medical Human Research Ethics Committee (HREC, #21786) and all participants provided prior written informed consent.

### 2.2 Autoantibody profiling

Antibody responses were analysed using the OmicsArray™ Antigen Microarray Profiling Services by GeneCopoeia (Rockville, Md). IgG specific responses to antigen targets on the Human Coronavirus-associated Autoimmunity (PA012) array were measured. Experimental protocol and data processing were provided by GeneCopoeia and were as follows:

#### 2.2.1 Experimental protocol

Array slides were blocked (room temperature (RT), 30 minutes) and washed 2x with phosphate buffered saline solution-Tween-20 (PBST, 5 minutes). Plasma samples diluted in PBST were added to wells of the slide (RT, 1 hour). After sample incubation arrays were washed with PBST, followed by blocking buffer, then PBST once more for 5 minutes each. After washing, anti-human IgG (Cy3-conjugated) antibodies (1:1000 in PBST) were added and incubated at RT for 1 hour. After incubation, arrays were washed with PBST (3x), PBS (2x) and finally nuclease-free water (2x), for 5 minutes per wash, before being spun down. From the addition of plasma samples, all incubation steps were performed on a shaker.

#### 2.2.2 Array image capture and data processing

Fluorescent signals were acquired using the GenePix 4000B microarray scanner, using 532nm channel to scan Cy3 fluorescence. To obtain the raw data, including foreground and background signals, and the signal to noise ratio (SNR), fluorescent signal was analysed using the GenePix™Pro v7.0 software. The net fluorescence intensity (NFI), representing the foreground median minus the background median, was calculated and the SNR and flags used to filter data. The net fluorescent value was calculated by subtracting the value of the PBS control. Robust linear model (RLM) normalization was performed to normalize the NFI of each sample (represented as NSI-Nor).

### 2.3 Statistical analysis

Antibody responses were considered positive if the NSI-Nor value was greater than the average plus three standard deviations of the negatives. Initial screening identified 9 uninfected control donors who were positive to at least one SARS-CoV-2 antigen. These were excluded from further analysis and were not included in the results section (final uninfected control cohort, n=21). Statistical significance was assessed using GraphPad Prism (v9.3.1). Where indicated in figure legends, NSI-Nor values were log transformed and normality distribution tested by the Anderson-Darling test, prior to assessing significance. For correlation analysis, Pearson’s correlation analysis was performed on log transformed NSI-Nor values and Spearman’s correlation on NSI-Nor values, where indicated. A heatmap was created in R Studio (2022.02.01), using the pretty heatmaps (‘pheatmap’) package.

## 3 Results

### 3.1 Clinical characteristics of cohort

In the present study, plasma samples were collected from healthcare workers, who were either previously SARS-CoV-2 PCR negative (n=21) or PCR positive (n=31), 8 months post an outbreak (**Table 1**). The COVID-19 convalescent cohort consisted of 25 females (80.6%) and 6 males (19.4%) with a median age of 48 years (range: 28-66 years). Additional clinical information such as co-morbidities for COVID-19 and other medical conditions were available for 26 individuals. Amongst these, 10 (38.5%) recorded at least one co-morbidity, including one with diabetes. Additionally, 3 (11.5%) individuals reported other medical conditions. Within the PCR negative cohort, 16 individuals (76.2%) were female, and the median age was 50 years (range: 31-65 years). COVID-19 co-morbidities and other medical conditions were available for 17 individuals, seven of whom, reported the presence of a COVID-19 co-morbidity, including one with diabetes. Four people additionally recorded other medical conditions.

**Table 1:** Clinical characteristics of study cohort.

		COVID-19 Convalescent (n=31)	Negatives (n=21)
Gender		F (25, 80.6%) M (6, 19.4%)	F (16, 76.2%) M (5, 23.8%)
Age Median (range)		48 (28-66)	50 (31-65)
Co-morbidities		38.5% (10/26)	41.2% (7/17)
	Asthma	3.85 (1/26)	5.9% (1/17)
	Chronic respiratory disease (excluding asthma)	0	0
	Heart disease (excluding high blood pressure)	3.85 (1/26)	5.9% (1/17)
	High blood pressure	0	5.9% (1/17)
	Immunosuppressive condition/therapy	3.85 (1/26)	0
	Diabetes	3.85 (1/26)	5.9% (1/17)
	Obesity (BMI >30 kg/m^2^)	23.1% (6/26)	17.6% (3/17)
	Liver disease	0	0
	Kidney disease	0	0
	Neurological disorder	0	5.9% (1/17)
	Pregnant during the period 20th March to 13th April 2020	0	5.9% (1/17)
Other medical conditions		11.5% (3/26)	23.5% (4/17)

Where information was available, those who tested positive for COVID-19 also self-reported the degree of their symptom severity and the symptoms they had during the outbreak period, where symptom severity was defined as mild (able to perform usual daily activities), moderate (decreased ability to conduct usual activities) or severe (unable to conduct usual daily activities and/or admitted to hospital for care) (**Table 2**). Additionally, they indicated whether they felt back to normal or not since their infection. Most of the present cohort had either mild (40%) or moderate (43.3%) symptom severity, covering a range of symptoms. The most common symptom was a headache (73.3%), followed by altered sense of taste or smell (56.7%). 16 individuals within the cohort (55.2%) noted that they felt back normal post their infection period.

**Table 2:** Characteristics of COVID-19 severity, symptoms, and recovery in COVID-19 convalescent cohort.

		
Degree of symptom severity	Number (n=30)	%
Mild	12	40.0
Moderate	13	43.3
Severe	5	16.7
Symptoms	Number (n=30)	%
Headache	22	73.3
Altered sense of taste or smell	17	56.7
Muscular Pain	15	50.0
Shortness of breath	15	50.0
Sore throat	15	50.0
Runny nose	14	46.7
Cough	13	43.3
Joint Pain	13	43.3
Fever	12	40.0
Diarrhoea	9	30.0
Chest pain	8	26.7
Irritability/confusion	8	26.7
Nausea/vomiting	8	26.7
Abdominal Pain	5	16.7
Other	2	6.7
No symptoms	0	0
Feel ‘normal’ post-infection	Number (n=29)	%
Yes	16	55.2
No/Unsure	13	44.8

### 3.2 Anti-spike and anti-nucleoprotein IgG responses remain high up to eight months post initial exposure

**Figure 1:**
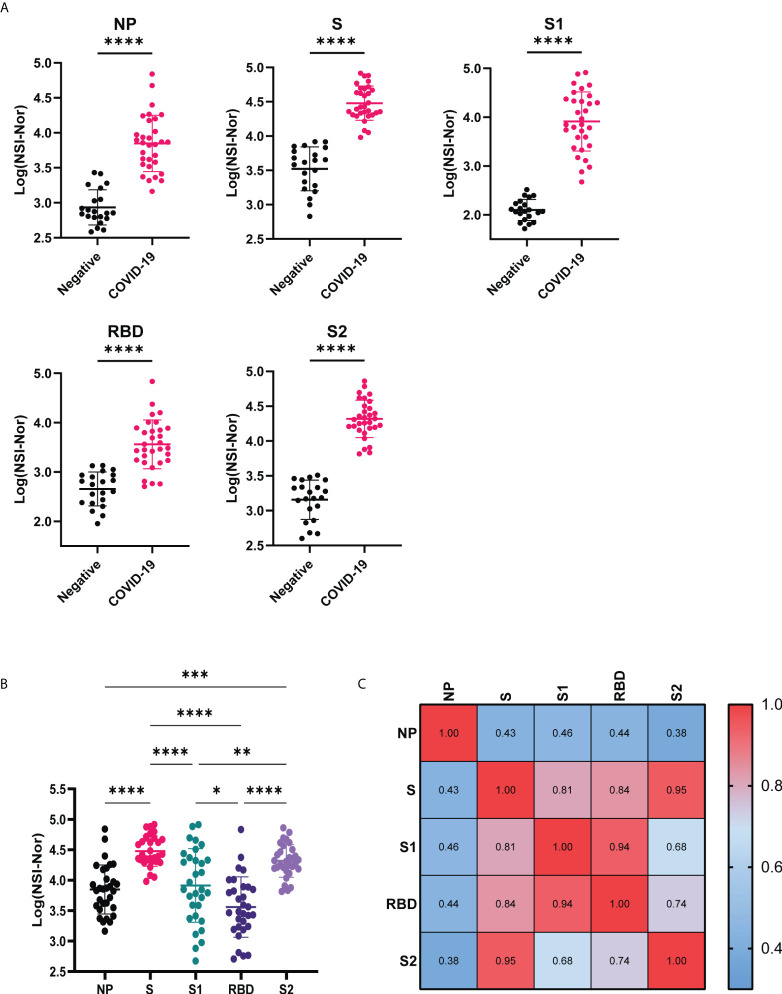
Anti-SARS-CoV-2 IgG responses to Nucleoprotein, Spike and Spike subunits. **(A)** Comparison of IgG-specific antibody responses against the SARS-CoV-2 Nucleoprotein (NP), Spike (S) and spike subunits S1, S2 and Receptor Binding Domain (RBD) between COVID-19 negative and convalescent plasma samples. **(B)** Comparison of IgG antibody responses to the different SARS-CoV-2 antigens within the convalescent group. **(C)** Pearson R correlation of anti-SARS-CoV-2 responses. Data shown as log transformed NSI-Nor values with mean ± standard deviation, and statistical significance assessed with unpaired T test **(A)** and Tukey’s multiple comparisons test **(B)**, where * p <0.05, ** p <0.01, *** p <0.0005, **** p <0.0001.

IgG antibody responses to five SARS-CoV-2 antigens, Nucleoprotein (NP), whole Spike (S), Spike S1 (S1), Receptor Binding Domain (RBD) and Spike S2 (S2), were measured and compared between the COVID-19 negative and convalescent cohorts (**Figure 1A**). Confirming previous reports of lasting antibody responses up to 12 months post infection (9, 10), a significant increase of antibody levels to the NP, S and S subunits was found in the COVID-19 convalescent cohort in comparison to uninfected individuals. We then compared the responses to each of the SARS-CoV-2 targets within the convalescent group (**Figure 1B**). A greater spread of responses was observed to NP, S1 and RBD, suggesting a potential decrease over time, in comparison to anti-S and -S2 responses, which remained high. Pearson R correlation analysis was performed to identify how the responses were related to each of the antigens (**Figure 1C**). Strong positive correlations were identified between the anti-S and S subunits. The strongest correlation was between S and S2 (r=0.95), indicating that the lasting antibody responses to the spike protein are most likely to be targeting the S2 region. Similarly, the anti-S1 and anti-RBD regions were strongly correlated (r=0.94) indicating the RBD region as the key targeting region within the S1 segment of S.

**Figure 2:**
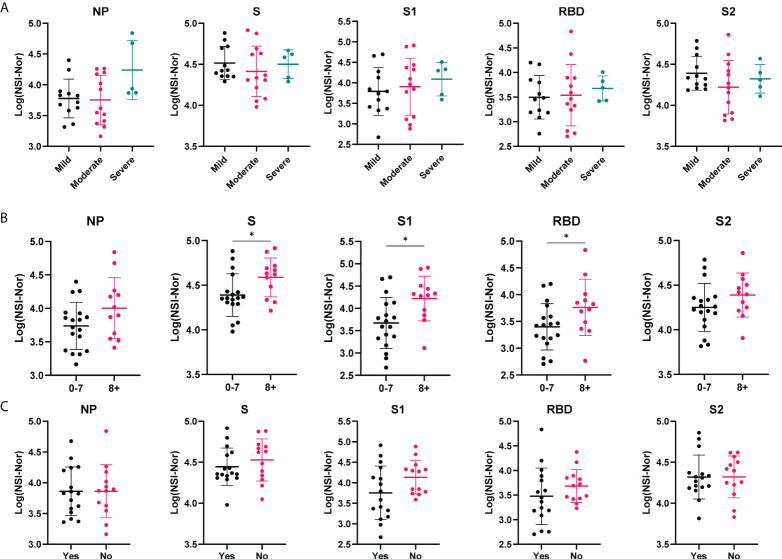
Antibody responses to SARS-CoV-2 antigens according to clinical characteristics. Anti-SARS-CoV-2 antibody responses to Nucleoprotein (NP), Spike (S) and spike subunits S1, S2 and Receptor Binding Domain (RBD), in the COVID-19 cohort based on self-reported categories. **(A)** Symptom severity as mild (n=12), moderate (n=13) or severe (n=5). **(B)** Number of symptoms experienced, 0-7 (n= 18) or 8+ (n=12). **(C)** whether ‘Yes’ they reported to feel normal post-infection (n=16) or ‘No’ they did not (n=13). Data shown as log transformed NSI-Nor values, with the mean ± standard deviation. Significance (*p<0.05) was assessed with the Kruskal-Wallis test **(A)**, unpaired T test **(B)** and Mann-Whiney test **(C)**.

To investigate whether clinical characteristics impacted lasting antibody levels to SARS-CoV-2, we divided the COVID-19 convalescent group based on the i) severity of their symptoms, ii) the number of symptoms experienced during the outbreak period and iii) whether they reported to feel ‘normal’ again post infection (**Figure 2**). To all five targets (NP, S, S1, RBD and S2) there were no differences in antibody responses among those experiencing mild, moderate or severe symptoms (**Figure 2A**). In comparison, higher titres of antibodies to SARS-CoV-2 antigens were found among those who self-reported a greater number of symptoms (8+) to those who reported less (0-7), with a significant increase to the S (*p=*0.029), S1 (*p=*0.012) and RBD (*p=*0.049) antigens (**Figure 2B**). As with symptom severity, no differences were found between those who reported feeling normal, or not, post infection (**Figure 2C**).

### 3.3 Individuals infected with earlier variants of the SARS-CoV-2 virus have low levels of responses to the Omicron variant spike protein

**Figure 3:**
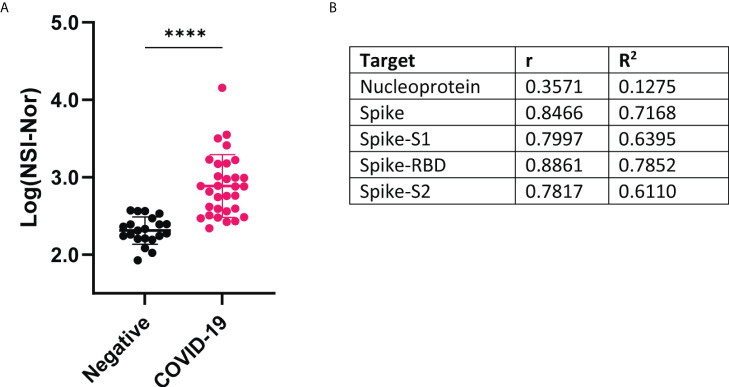
IgG antibody responses to Omicron variant spike protein and correlation with Wuhan-Hu-1 antigens **(A)** Comparison of antibody responses to Omicron spike protein between COVID-19 convalescent and negative groups. Data shown as log transformed NSI-Nor values and unpaired T tested performed, **** p <0.0001 **(B)** Pearson R correlation results between anti-Omicron spike antibody response and anti-original Wuhan-specific targets.

Omicron (B.1.1.529) is the latest variant of concern, already consisting of multiple sub-lineages (33). IgG responses to the Omicron spike protein were measured and found to be significantly increased in the COVID-19 convalescent samples, suggesting cross-reactivity between infection with an earlier variant and the Omicron variant (**Figure 3A**). To identify how the level of anti-Omicron spike responses compared to the level of the anti-Wuhan-Hu-1 sequence targets, Pearson R correlation analysis was performed (**Figure 3B**). A low level of positive correlation was found between the anti-S Omicron response and anti-NP responses, r=0.3561 and R^2 =^ 0.1275. In comparison, a strong positive correlation was observed between the IgG responses to Omicron spike and each of the Wuhan-Hu-1 spike targets, with the strongest correlation being to the RBD region (r=0.8861, R^2 =^ 0.7852). Given anti-RBD responses are lower than the anti-S, -S1 and -S2 responses, this suggests a low level of cross-reactivity occurring.

**Figure 4:**
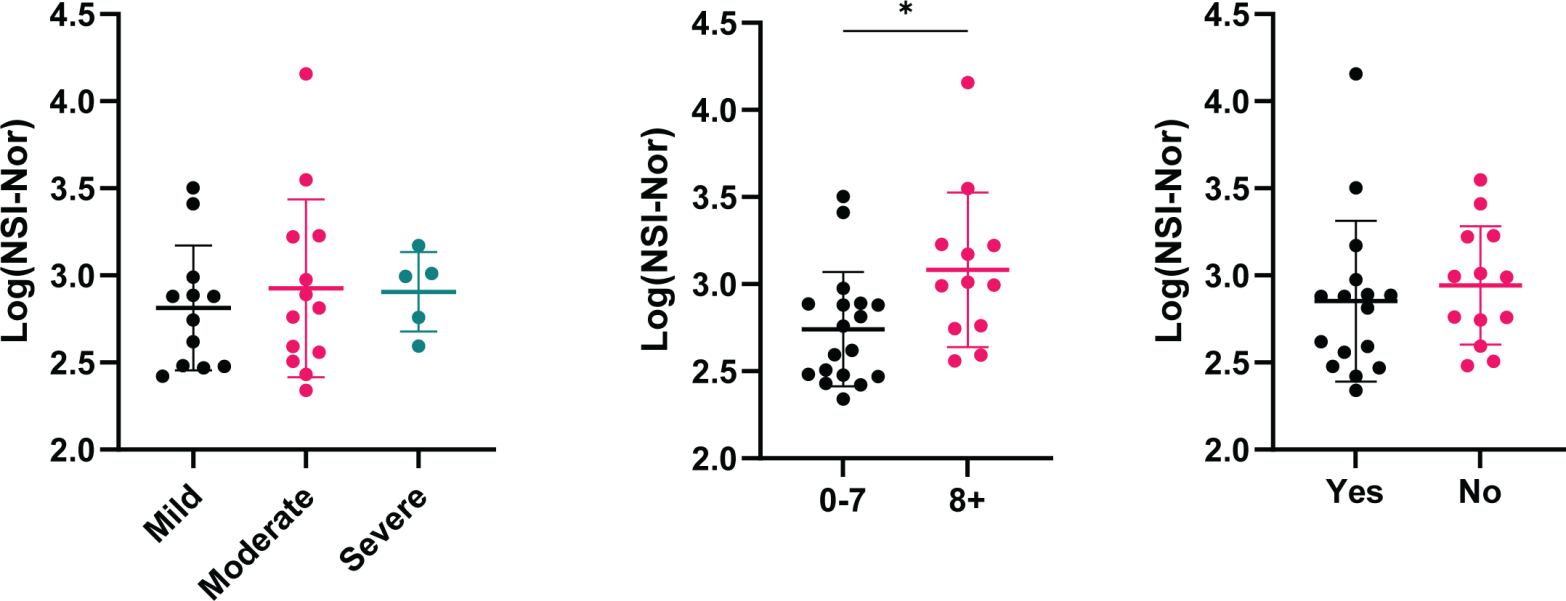
Cross-reactive antibody responses to Omicron spike according to clinical characteristics. IgG antibody responses to Omicron spike protein based on self-reported categories (Left) Symptom severity as mild (n=12), moderate (n=13) or severe (n=5). (Middle) Number of symptoms experienced, 0-7 (n=18) or 8+ (n=12), p=0.015. (Right) Whether ‘Yes’ (n=16) or 'No' (n=13) they reported to feel back to normal, or not, post infection. Data shown as log transformed NSI-Nor values, with the mean ± standard deviation. Significance (*p < 0.05) was assessed with the Kruskal-Wallis test and Mann-Whitney test, respectively.

With the presence of cross-reactivity to the Omicron spike protein, we investigated whether patient clinical characteristics impacted the level of cross-reactive responses. While no differences were found between symptom severity and feeling ‘normal’ post infection, the anti-Omicron Spike responses were significantly higher in those who experienced more symptoms (**Figure 4**).

### 3.4 Autoantibodies post COVID-19

**Figure 5:**
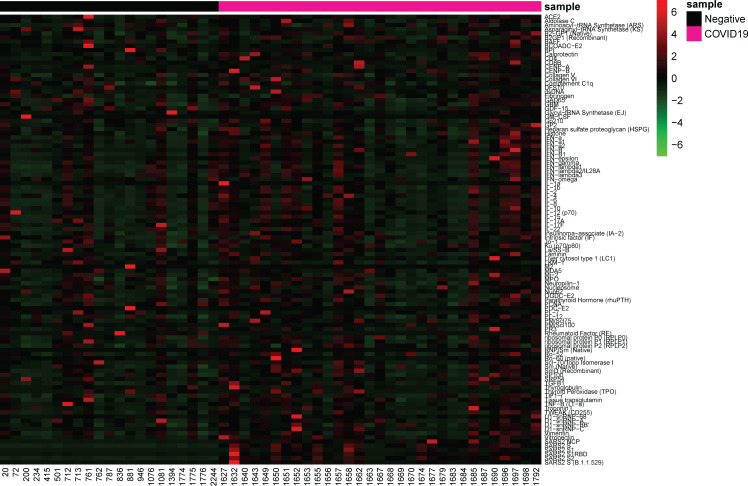
Antibody responses to SARS-CoV-2 antigens and autoantigens. Heatmap depicting NSI-Nor values of IgG antibodies to SARS-CoV-2 antigens (n=6) and autoantigens (n=102). COVID-19 negative (n=21) and COVID-19 convalescent (n=31) individuals grouped along the x-axis. Autoantigens listed alphabetically along y-axis, and SARS-CoV-2 antigens at the bottom.

**Figure 6:**
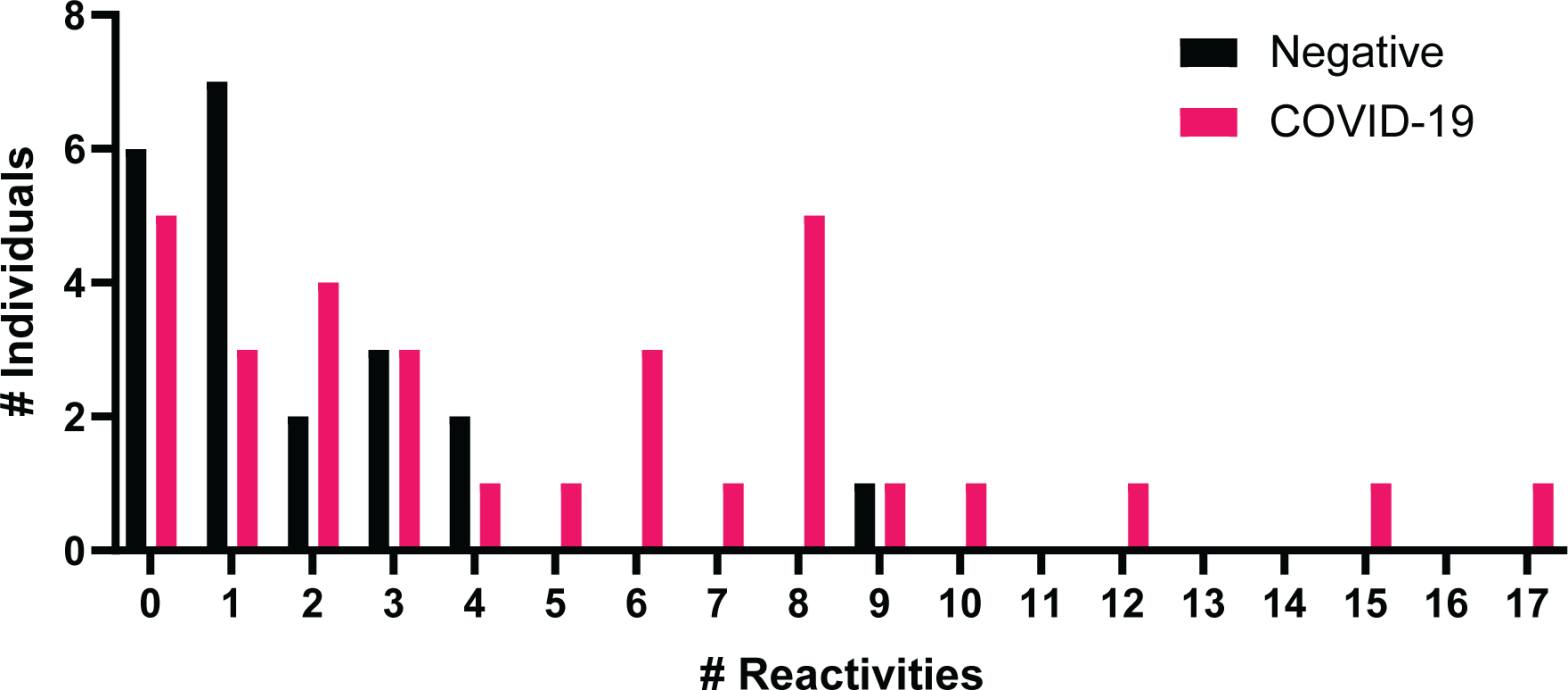
Number of positive autoantibody reactivities in COVID-19 negative and convalescent individuals. The number of positive autoantibodies reactivities in COVID-19 negative and convalescent individuals. Positive considered as NSI-Nor values at or above the average plus three standard deviations of the COVID-19 negative cohort.

Autoantibodies towards a variety of antigens have been shown in severe COVID-19 patients in early stages of infection (28, 30) and in convalescent samples (5, 29). IgG specific autoantibodies to 102 known autoantigens were measured in the COVID-19 negative and convalescent cohorts to identify whether COVID-19 results in a potential increase in long-term autoreactivity (**Figure 5**). A range of reactivities was found to each of the antigens amongst both the COVID-19 convalescent and negative groups. Using the applied average of negatives plus three standard deviations cut-off, the number of positive reactivities in individuals was identified (**Figure 6**). 26/31 (83.9%) convalescent COVID-19 individuals had a positive reactivity to at least one autoantigen, covering 63/102 (61.8%) autoantigens (**Supplementary Table 1**). Although negative for SARS-CoV-2 responses, it was also found that 15/21 (71%) COVID-19 negative individuals were positive for at least one autoantigen (majority between 0-4 reactivities). One COVID-19 negative individual had positive autoreactivities to nine targets, which could be a normal state or indicate a break in immune tolerance and some underlying cause of immune dysfunction. Amongst the negative cohort, positive reactivities were identified to 37/102 (36.2%) autoantigens. Almost all autoantigens were positive in only one individual, except for Asparaginyl-tRNA Synthetase (KS), found to be positive in two individuals. When comparing the number of reactivities between the COVID-19 convalescent and negative groups, those who had been infected with SARS-CoV-2 showed a greater range of the number of positive autoantibody reactivities (**Figure 4**). On average, the COVID-19 convalescent group had 5.1 positive reactivities in comparison to the negative cohort’s 1.8, highlighting a 2.9-fold increase of the number of autoreactive antibodies post COVID-19.

**Figure 7:**
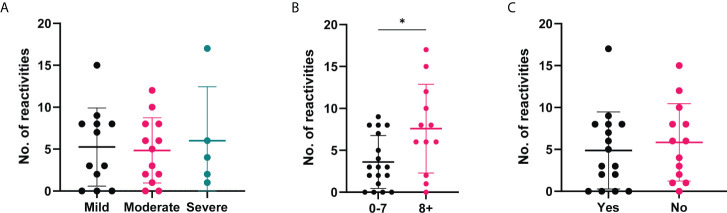
Number of positive autoantibody reactivities in COVID-19 convalescent cases based on self-reported symptoms and recovery. The number of positive autoantibodies according to self-reported categories. **(A)** Symptom severity reported as mild (n=12), moderate (n=13) or severe (n=5). **(B)** Number of symptoms experienced, 0-7 (n=18) or 8+ (n=12), p=0.045. **(C)** Whether 'Yes' they felt normal post-infection (n=16) or 'No' they did not (n=13). Data shown as log transformed NSI-Nor values, with the mean ± standard deviation. Significance (*p < 0.05) was assessed with the Kruskal-Wallis test, Mann-Whitney and Unpaired T test, respectively.

Due to the range of positive autoantibody responses identified in the COVID-19 convalescent cohort, we were interested in whether the number of positive reactivities was associated with the clinical severity or outcomes (**Figure 7**). While no significant differences were observed based on disease severity (**Figure 7A**), those who reported to experience more symptoms during the outbreak period were found to have more positive autoantibody reactivities in comparison to those with less symptoms (**Figure 7B**). No differences in the number of positive autoantibodies were found between those who reported to feel normal post infection and those who did not (**Figure 7C**).

### 3.5 Identification of some of the top autoantigen targets in the COVID-19 convalescent cohort

In addition to exploring the number of autoantibody reactivities in an individual, we further explored whether there were common autoantigen targets in convalescent COVID-19 individuals. Of the 63 autoantigens recognised within the cohort, 13 of these were found in 4 individuals or more (**Table 3**). The most common were autoantibodies to calprotectin, identified in 7 individuals (22.58%).

**Table 3:** Autoantigens with the highest positive reactivities in COVID-19 convalescent individuals.

Antigen	Number of Individuals	% Positive
Calprotectin	7	22.58
CD4	6	19.35
B2GP1 (Recombinant)	6	19.35
IFN-α2	6	19.35
RNP/Sm (Native)	6	19.35
CENP-B	5	16.13
U1-snRNP-68	5	16.13
IFN-α	4	12.90
PM/Scl75	4	12.90
Vitronectin	4	12.90
Histone	4	12.90
IFN-β1	4	12.90
SmD (Recombinant)	4	12.90

**Figure 8:**
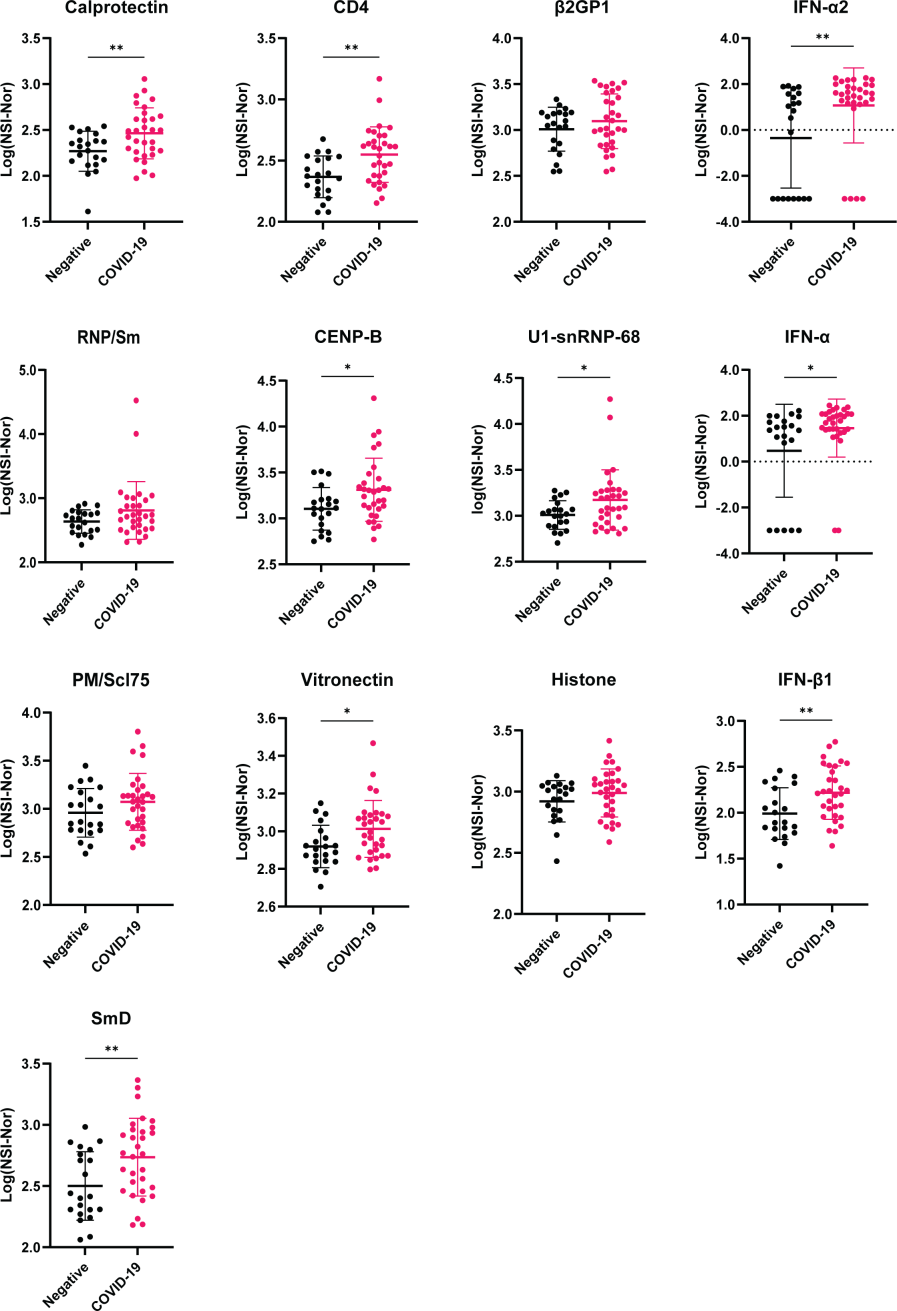
Comparison of antibody responses between COVID-19 negative and convalescent individuals for targets with highest number of positive reactivities. Plots of the 13 autoantigens with the highest number of positive COVID-19 convalescent individuals. Following transformation, normality was tested by the Anderson-Darling test prior assessing significance using either unpaired T-test or Mann-Whitney, for normal and non-normal distributed data, respectively. *p<0.05, ** p< 0.01. Data shown as log transformed NSI-Nor values with mean ± standard deviation.

Using these top antigens based on the number of ‘positive’ reactivities as potential autoantigens of interest, we compared the antibody responses between the COVID-19 convalescent and negative cohorts (**Figure 8**). None of the 13 antigens identified in **Table 1** were amongst the 37 antigens with a positive reactivity in the negative cohort, suggesting that these targets may be specific to post COVID-19. Of these autoantigens, 9/13 showed a significant difference between the groups, highlighting a potential association of autoantibodies to these autoantigens and COVID-19 infection. No significant difference was observed between the groups for Recombinant β2GP1, RNP/Sm (Native), PM/SCL75 and Histone, highlighting the variation of autoantigens targeted within individuals with COVID-19, as reported previously (30).

**Figure 9:**
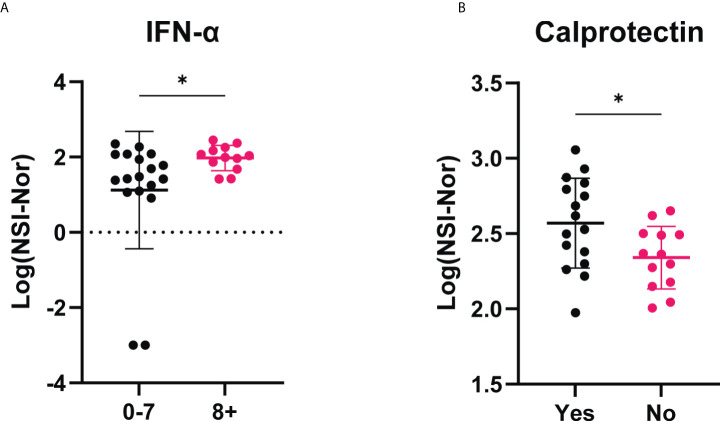
IgG antibody responses to IFN-α and calprotectin among COVID-19 convalescent individuals based on self-reported clinical characteristics. **(A)** Anti-IFN-α autoantibody responses compared between those who experienced less (0-7, n=18) or more (8+, n=12) symptoms during the initial outbreak period. **(B)** Anti-calprotectin autoantibodies according to self-reported ‘Yes’ they felt normal (n=16) or ‘No’ they did not feel normal (n=13) eight months post infection period. Data shown as log transformed NSI-Nor values with the mean ± standard deviation. Following normality test, significance (*p < 0.05) assessed using **(A)** Mann-Whitney test, p=0.030, and **(B)** unpaired T test, p=0.027.

Given acute clinical severity has been associated with the presence of autoantibodies, such as type I interferons (27), we furthered investigated whether each of the 13 identified autoantigens (**Table 3**/**Figure 8**), specific to the COVID-19 convalescent cohort, had an association with the self-reported clinical symptoms and recovery (**Supplementary Figures 1–3**). No differences of responses to each of the autoantigens was found based on symptom severity (**Supplementary Figure 1**). Comparisons based on the number of symptoms experienced or whether individuals reported to feel ‘normal’ or not post infection both identified one autoantigen with a difference between the groups compared (**Figure 9**). Autoantibodies to IFN-α were found to be significantly higher in individuals who reported a greater number of symptoms experienced during the initial outbreak (**Figure 9A**), whereas higher anti-calprotectin antibodies were identified in those who reported to feel normal again (**Figure 9B**).

### 3.6 Level of anti-Spike antibodies is associated with the number of autoantibody reactivities

To explore whether there was a correlation between the anti-SARS-CoV-2 responses and autoantigen responses, Spearman R correlation was performed between all targets on the arrays (**Supplementary Table 2**). The strongest positive correlations were found between the autoantibody responses, particularly within the anti-cytokine antibodies. Using 0.5 as a cut-off for moderate positive correlation, antibody responses to 11 autoantigens were found to correlate with the antibody responses to at least one SARS-CoV-2 antigen (**Table 4**). The antibody responses to NP did not correlate with autoantibodies. In contrast, it was found that anti-Omicron spike responses had a moderate correlation with the most autoantibodies (10/11). Amongst the 11 autoantigens, two targets, SmD (recombinant) and Thyroglobulin had positive correlations with all SARS-CoV-2 spike targets.

**Table 4:** Moderate correlation between anti-SARS-CoV-2 responses and autoantibody responses.

	NP	S	S1	RBD	S2	Omicron S
**CENP-A**	**-**	–	–	–	–	0.52
**PDC-E2**	**-**	–	–	–	–	0.50
**PR3**	**-**	–	–	–	–	0.50
**Ribosomal protein P1 (RPLP1)**	**-**	–	–	–	–	0.57
**Ro-52**	**-**	–	–	0.50	–	–
**SmD (Recombinant)**	**-**	0.55	0.50	0.57	0.51	0.69
**TGFβ1**	**-**	–	–	0.54	–	0.62
**Thyroglobulin**	**-**	0.56	0.59	0.59	0.51	0.70
**Thyroid Peroxidase (TPO)**	**-**	0.51	–	0.53	–	0.52
**LTA (TNF-β)**	**-**	–	–	0.56	–	0.61
**Vitronectin**	**-**	–	–	–	–	0.55
**# correlated**	0	3	2	6	2	10

**Figure 10:**
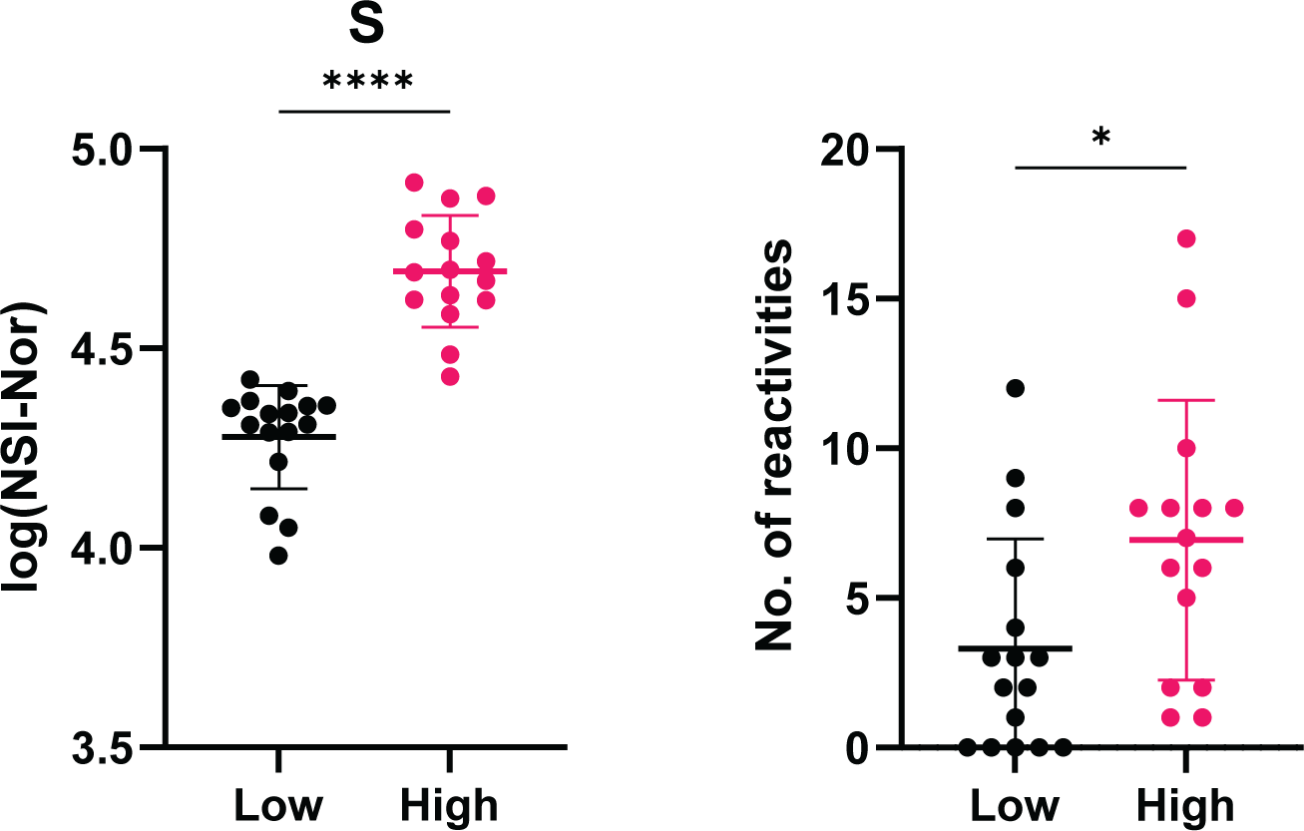
Association between level of anti-Spike antibody responses and the number of positive autoantibody reactivities. COVID-19 convalescent individuals (n=31) divided into high and low anti-S using the median of responses as the dividing point. The number of autoantibody reactivities within the individuals split into the corresponding high or low group. Normality tested using the Anderson-Darling test prior assessing significance using the Mann-Whitney test. *p<0.05, **** p< 0.0001. Data shown as mean ± standard deviation.

As a range of the number of positive reactivities could be found within the COVID-19 convalescent group (0-17, **Figure 6**), we explored whether there was an association between the anti-SARS-CoV-2 responses and the number of positive reactivities. Using the median as a cut-off, the convalescent cohort was divided into high and low anti-SARS-CoV-2 responders and the number of reactivities per individual split accordingly (**Supplementary Figure 4**). In doing so, a significant difference was identified between the number of reactivities in high and low anti-S responders (**Figure 10**). This suggests an association between the levels of anti-S antibodies and the presence of autoantibodies.

## 4 Discussion

Viral infections have known associations to autoimmunity [reviewed by Smatti et al. (34)] and autoantibodies have been reported in COVID-19 patients (25–30). Using microarrays consisting of SARS-CoV-2 antigens and 102 autoantigens, we sought to explore the presence of anti-SARS-CoV-2 antibodies, including cross-reactivity to Omicron, and autoantibodies eight months after infection. Antibody responses to the SARS-CoV-2 NP, S and S subunits S1, S2 and RBD remained high in most convalescent samples, in comparison to the uninfected control group. Correlation analysis showed lasting antibody responses to the whole spike protein are specifically to the S2 subunit, whereas within the S1 subunit, it is the RBD region which is highly antigenic. Antibody cross-reactivity was additionally found to the Omicron variant in individuals infected with the Wuhan-Hu-1 variant. A range of autoantibodies were found in both COVID-19 convalescent and uninfected individuals, with a greater spread of the number of positive reactivities found in the convalescent cohort. Higher titres of anti-SARS-CoV-2 responses, positive autoantibody reactivities, and autoantibodies to IFN-α were found to be associated with those who experienced more symptoms during the initial outbreak period. While anti-SARS-CoV-2 responses and autoantibody positivity was not associated with feeling ‘back to normal’ eight months post infection, we identified one autoantigen of interest, calprotectin, which was found to have higher autoantibody responses among those who reported feeling back to normal. Finally, we found an association between the number of positive autoantibody reactivities and the level of anti-S antibodies.

Antibodies play an important role in the anti-viral response, particularly in neutralisation and immune memory. Given this, understanding their persistence is important in the context of responses following re-exposure and/or vaccination. Studies have reported that antibodies to the SARS-CoV-2 spike protein and nucleoprotein can persist for over 11 months post infection (12, 35). In the present study, antibody responses were measured to five SARS-CoV-2 antigens within the NP and S proteins and while responses to NP, S1 and RBD were lower in some individuals, anti-S and -S2 were consistently high. This trend is consistent with other studies, which report the similar lower or sustained responses, within 2-12 months post COVID-19 (9, 14, 35, 36). Correlation analysis between the anti-SARS-CoV-2 responses indicated the similarity between responses to S and S2 and between S1 and RBD. To our knowledge, similar correlations between the anti-SARS-CoV-2 antigens responses has not been done, however anti-S1 responses have been shown to correlate with neutralisation (12, 36) indicating the RBD region is the main antigenic target of S1, as observed here. The association between disease severity and lasting anti-SARS-CoV-2 responses post infection is inconsistent, with some studies reporting a correlation with disease severity (37) and higher responses in severe cases (17, 18), and others reporting no differences (19, 20). In the present study, we found anti-SARS-CoV-2 responses did not differ according to the self-reported symptom severity. However, this may be due to low sample numbers within each group, especially the 'severe' group, or because a different measure for severe disease was used, in which participants were unable to conduct usual daily activities and/or admitted to hospital for care. Additionally, anti-SARS-CoV-2 responses did not differ based on returning to ‘normal’ or not. This is consistent with other studies comparing anti-SARS-CoV-2 responses in individuals with or without long-COVID at four (21), six (16) and 12 (8) months. Indeed, rather than disease severity, it was found that individuals who reported a greater number of symptoms, experienced during the initial outbreak period, had significantly higher anti-S, -S1 and -RBD antibody levels, indicating an association between symptoms and antibody responses.

In the present study, COVID-19 convalescent individuals, exposed during the first SARS-CoV-2 (Wuhan-hu-1) outbreak in Tasmania, Australia, in 2020, prior to vaccine development, were found to have antibody responses to the Omicron spike protein, indicating immune cross-reactivity. As a highly vaccinated society, with over 12 billion doses administered worldwide (as of 25th July, 2022) (38), several studies have reported that vaccination-induced protection against Omicron requires three doses for neutralising protection (23, 39). Pre-exposure to other variants alone has shown inability to neutralise the Omicron variant (23). However, some neutralisation of Omicron was found in individuals who had pre-exposure to either D614G variant or Epsilon (B.1.429) then two mRNA vaccine doses. While we did not measure neutralisation, the presence of cross-reactive antibodies, from infection alone, indicates pre-immunity from the prior exposure which can be boosted by vaccination to offer protection against Omicron and possibly future emerging variants.

Autoantibodies have been reported in acute COVID-19 (25–27, 30) and some post-COVID-19 cohorts (5, 29). Here, autoantibodies to a range of antigens in COVID-19 individuals, eight months post infection, and in uninfected individuals were found. Autoimmune responses can be found in healthy individuals and could explain autoantibodies in the uninfected group, given only one individual reported a known autoimmune history (40, 41). Indeed, a recent study investigated the “autoantibodyome” amongst healthy and disease cohorts and identified 77 common autoantibodies within the healthy individuals (42). Amongst the thousands of human proteins measured against, none of the 102 disease specific autoantigens measured in this study were among the 77 common targets. Rather, given high levels of autoantibodies have been reported in COVID-19, in comparison to healthy controls, through identifying the number of positive reactivities, we found that antigen targets in the negative group were recognised by single individuals, whereas there were common antigens amongst the COVID-19 convalescent cohort.

To our knowledge, three antigens that were found to be autoantigens in multiple COVID-19 convalescent individuals, namely calprotectin, CD4 and vitronectin, have not been reported as autoantigens of interest either during acute or post-COVID-19. Of these, calprotectin was recognised by the highest number of individuals in our cohort (7/31, 22.58%) and was the only autoantigen to show an association with clinical recovery, specifically it was higher in those reporting feeling ‘normal’ post-infection. Calprotectin is a calcium binding protein produced by activated monocytes and neutrophils (43). Increased levels have been found in inflammatory bowel disease (44), rheumatic diseases (45) and myasthenia gravis (46). In COVID-19, calprotectin has been reported in several studies to be increased and associated with severe disease (47, 48). This suggests the autoantibodies could be induced by increased antigen presence and given anti-calprotectin was significantly higher in individuals reporting being back to normal post-infection, future studies may address whether these autoantibodies may provide functional protection. Interestingly, to our knowledge, IgG specific autoantibodies to calprotectin have only been reported in one other study (5). In that study, using a less stringent positive cut off than in the present study, 3% (3/100) of a long-COVID cohort were anti-calprotectin positive. Given this low positive rate in a long-COVID cohort, it supports a hypothesis that the presence of anti-calprotectin may indeed be protective. However, further studies with larger cohorts comparing individuals with and without long-COVID will provide further insights into the role of these autoantibodies.

Studies using microarrays have reported the presence of autoantibodies in acute COVID-19 individuals who are either hospitalised (30) or exhibit a range of clinical severity (28). While different targets were highlighted within each study, both note a high prevalence of anti-cytokine autoantibodies including to those of type 1 IFNs. Within our COVID-19 convalescent cohort, we found positive reactivities to IFN-α2, IFN-α and IFN-β1 in 19.35%, 12.90% and 12.90% respectively. Interestingly, while anti-IFN’s have been reported to be associated with acute severe disease (27), we identified increased titres to IFN-α in those who reported a greater number of symptoms experienced during the outbreak. Also found in our top reactivities were β-2 glycoprotein I (β2GP1) and CENP-B. β2GP1, positive in 19.35% of the present cohort, has previously been reported in acute COVID-19 (26, 30). In contrast, CENP-B, found in 16.13% of the present COVID-19 convalescent cohort, and higher than the COVID-19 negative cohort, has previously been reported to be infrequent in early hospitalisation (30). Given these autoantibody reactivities were identified in convalescent samples, it suggests that autoantibodies raised during acute infection, can last up to 8 months post initial exposure. However, without earlier time points to track changes, this cannot be confirmed. Another study has highlighted that autoantibodies post-COVID-19 can last up to 6 months in a sex-dependent manner, no matter the initial disease severity (29). Lasting autoantibodies may be relevant and open questions about their role in post-COVID-19 syndrome/long-COVID. Indeed, reports of ANAs (8) and functional autoantibodies to G-protein coupled receptors (7) have been reported in those with long-COVID as well as recently emerging literature highlighting persistent autoimmunity (6) and a range of autoreactivities (5). However, except for anti-calprotectin, we found no differences in levels of antibody responses to our top 13 autoantigens between those who reported to feel normal or not post-infection. Therefore, further studies, in larger cohorts, are required to understand autoimmunity, and autoantigens, both during and post COVID-19 to give further insights into the aetiology of long-COVID and potential treatments.

The magnitude of antibody responses to SARS-CoV-2 have been shown to correlate with COVID-19 disease severity, where higher antibody responses develop during acute disease in more severe cases (13, 15). Here, we correlated the anti-SARS-CoV-2 responses with the autoantigen responses and identified a range of degrees of correlation, with the majority showing weak to moderate positive correlations. Similar anti-SARS-CoV-2 and autoantibody correlations have been shown in other study cohorts with anti-NP (29) and -S, -S1, -RBD and -S2 (5). Correlations between anti-NP and autoantibodies were reported to be non-significant (29) or few (5), consistent with our findings of no positive correlation using the 0.5 cut-off. Interestingly, where anti-NP and autoantibody unadjusted correlation was performed, thyroid peroxidase (TPO) was one of nine targets reported to correlate (29). TPO was found to correlate with anti-S, -RBD and -S Omicron in our cohort but with anti-S, -S1, -RBD and -S2 in a third cohort of long-COVID individuals (5). Furthermore, similar correlation patterns were found between our cohort and the cohort of long-COVID individuals with positive correlations between anti-S/S subunits and thyroglobulin and TGF-β1 (5). In the long-COVID cohort, antibodies to these two targets also showed correlation with anti-NP. While amongst our top correlation antigens, CENP-A and LTA (TNF-β) were identified to correlate with anti-S Omicron, which consistent with the long-COVID cohort, there was no correlation with the original Wuhan-Hu-1 antigens. In contrast, antibody responses to SmD and LTA were found to correlate with responses to all Wuhan-Hu-1 and RBD antigens, respectively, but not to any target in the long-COVID cohort. In addition to these correlations, we found individuals with higher antibody responses to the spike protein, had an overall greater number of autoantibody reactivities, further indicating a connection between anti-SARS-CoV-2 responses and the presence of autoantibodies.

In the present study, antibody responses to SARS-CoV-2 antigens and a range of known autoantigens were measured in plasma samples from a cohort of healthcare workers exposed to Wuhan-Hu-1, in a single exposure event, eight months prior to blood collection. A range of autoantibody responses were identified, and a greater number of positive reactivities found within COVID-19 convalescent samples and more-so in those with higher anti-S responses. However, without analysing acute disease samples, or having pre-infection samples, conclusions about the rise and persistence of autoantibodies cannot be made. The identification of anti-calprotectin autoantibodies as potentially protective indicates a need to not only explore the induction of autoimmunity but to understand specific targets that may be involved in pathology or protection. Future analysis investigating the longitudinal autoantibody responses to antigens identified in this study and their correlation with disease severity and outcomes, may give insights into the roles of autoantibodies in long-COVID.

## Data availability statement

The original contributions presented in the study are included in the article/**Supplementary Material**. Further inquiries can be directed to the corresponding author.

## Ethics statement

The studies involving human participants were reviewed and approved by Tasmanian Health and Medical Human Research Ethics Committee. The patients/participants provided their written informed consent to participate in this study.

## Author contributions

Conceptualization, RM and MP; Participant recruitment and sample collection, FHJ, KLF, KJS, MM, NS, and SS; Methodology, RM, and MP; Investigation, RM; Resources, FJ, NS, KS, KLF and MP; Writing—original draft, review and editing, RM, SS, FHJ, KJS, NS, MM, KLF and MP; Supervision, MP. All authors have read and agreed to the published version of the manuscript

## Funding

MP is an NHMRC Research Fellow. MP and KLF receive NHMRC funding; KLF, MP and SS received funding from the Clifford Craig Foundation; RM is a recipient of an Australian Government Research Training Program Scholarship. This study was also supported by a philanthropic donation from Mrs Rebecca Round.

## Acknowledgments

The authors thank all the healthcare workers who consented to be part of the study. We also acknowledge the nurses who collected the bloods, Prof Lizzie Shires who supported the logistics of setting up the blood collection clinics and Dr Louise Parry who assisted with blood collections and recruitment.

## Conflict of interest

The authors declare that the research was conducted in the absence of any commercial or financial relationships that could be construed as a potential conflict of interest.

## Publisher’s note

All claims expressed in this article are solely those of the authors and do not necessarily represent those of their affiliated organizations, or those of the publisher, the editors and the reviewers. Any product that may be evaluated in this article, or claim that may be made by its manufacturer, is not guaranteed or endorsed by the publisher.

## References

[1] Huang C Wang Y Li X Ren L Zhao J Hu Y. Clinical features of patients infected with 2019 novel coronavirus in wuhan, China. Lancet (2020) 395(10223):497–. doi: 10.1016/S0140-6736(20)30183-5 PMC7159299 31986264

[2] Han H Xie L Liu R Yang J Liu F Wu K. Analysis of heart injury laboratory parameters in 273 covid-19 patients in one hospital in wuhan, China. J Med Virol (2020) 92(7):819–23. doi: 10.1002/jmv.25809 PMC7228305 32232979

[3] Nalleballe K Reddy Onteddu S Sharma R Dandu V Brown A Jasti M. Spectrum of neuropsychiatric manifestations in covid-19. Brain Behav Immun (2020) 88:71–4. doi: 10.1016/j.bbi.2020.06.020 PMC7297688 32561222

[4] Moody R Wilson K Flanagan KL Jaworowski A Plebanski M. Adaptive immunity and the risk of autoreactivity in covid-19. Int J Mol Sci (2021) 22(16):8965. doi: 10.3390/ijms22168965 34445670 PMC8396528

[5] Rojas M Rodríguez Y Acosta-Ampudia Y Monsalve DM Zhu C Li Q-Z. Autoimmunity is a hallmark of post-covid syndrome. J Trans Med (2022) 20(1):129. doi: 10.1186/s12967-022-03328-4 PMC8924736 35296346

[6] Acosta-Ampudia Y Monsalve DM Rojas M Rodríguez Y Zapata E Ramírez-Santana C. Persistent autoimmune activation and proinflammatory state in post-coronavirus disease 2019 syndrome. J Infect Dis (2022) 225:2155–62. doi: 10.1093/infdis/jiac017 PMC8903340 35079804

[7] Wallukat G Hohberger B Wenzel K Fürst J Schulze-Rothe S Wallukat A. Functional autoantibodies against G-protein coupled receptors in patients with persistent long-Covid-19 symptoms. J Transl Autoimmun (2021) 4:100100–. doi: 10.1016/j.jtauto.2021.100100 PMC8049853 33880442

[8] Seeßle J Waterboer T Hippchen T Simon J Kirchner M Lim A. Persistent symptoms in adult patients 1 year after coronavirus disease 2019 (Covid-19): A prospective cohort study. Clin Infect Dis (2021) 74(7):1191–8. doi: 10.1093/cid/ciab611 PMC8394862 34223884

[9] Masiá M Fernández-González M Telenti G Agulló V García JA Padilla S. Durable antibody response one year after hospitalization for covid-19: A longitudinal cohort study. J Autoimmun (2021) 123:102703–. doi: 10.1016/j.jaut.2021.102703 PMC8289631 34303083

[10] Li C Yu D Wu X Liang H Zhou Z Xie Y. Twelve-month specific igg response to sars-Cov-2 receptor-binding domain among covid-19 convalescent plasma donors in wuhan. Nat Commun (2021) 12(1):4144–. doi: 10.1038/s41467-021-24230-5 PMC8260809 34230476

[11] De Giorgi V West KA Henning AN Chen LN Holbrook MR Gross R. Naturally acquired sars-Cov-2 immunity persists for up to 11 months following infection. J Infect Dis (2021) 224(8):1294–304. doi: 10.1093/infdis/jiab295 PMC8195007 34089610

[12] Choe PG Kang CK Kim K-H Yi J Kim ES Park SW. Persistence of neutralizing antibody response up to 1 year after asymptomatic or symptomatic sars-Cov-2 infection. J Infect Dis (2021) 224(6):1097–9. doi: 10.1093/infdis/jiab339 34166506

[13] Plūme J Galvanovskis A Šmite S Romanchikova N Zayakin P Linē A. Early and strong antibody responses to sars-Cov-2 predict disease severity in covid-19 patients. J Trans Med (2022) 20(1):176. doi: 10.1186/s12967-022-03382-y PMC9012069 35428263

[14] Nguyen-Contant P Embong AK Kanagaiah P Chaves FA Yang H Branche AR. S protein-reactive igg and memory b cell production after human sars-Cov-2 infection includes broad reactivity to the S2 subunit. mBio (2020) 11(5):e01991–20. doi: 10.1128/mBio.01991-20 PMC7520599 32978311

[15] Lynch KL Whitman JD Lacanienta NP Beckerdite EW Kastner SA Shy BR. Magnitude and kinetics of anti–severe acute respiratory syndrome coronavirus 2 antibody responses and their relationship to disease severity. Clin Infect Dis (2021) 72(2):301–8. doi: 10.1093/cid/ciaa979 PMC7454426 33501951

[16] Ryan FJ Hope CM Masavuli MG Lynn MA Mekonnen ZA Yeow AEL. Long-term perturbation of the peripheral immune system months after sars-Cov-2 infection. BMC Med (2022) 20(1):26. doi: 10.1186/s12916-021-02228-6 35027067 PMC8758383

[17] Dan JM Mateus J Kato Y Hastie KM Yu ED Faliti CE. Immunological memory to sars-Cov-2 assessed for up to 8 months after infection. Science (2021) 371(6529):eabf4063. doi: 10.1126/science.abf4063 33408181 PMC7919858

[18] Horton DB Barrett ES Roy J Gennaro ML Andrews T Greenberg P. Determinants and dynamics of sars-Cov-2 infection in a diverse population: 6-month evaluation of a prospective cohort study. J Infect Dis (2021) 224(8):1345–56. doi: 10.1093/infdis/jiab411 PMC8436370 34387310

[19] Marklund E Leach S Axelsson H Nyström K Norder H Bemark M. Serum-igg responses to sars-Cov-2 after mild and severe covid-19 infection and analysis of igg non-responders. PLoS One (2020) 15(10):e0241104–e. doi: 10.1371/journal.pone.0241104 PMC7577439 33085715

[20] Ozgocer T Dagli ŞN Ceylan MR Disli F Ucar C Yildiz S. Analysis of long-term antibody response in covid-19 patients by symptoms grade, gender, age, bmi, and medication. J Med Virol (2022) 94(4):1412–8. doi: 10.1002/jmv.27452 PMC8662095 34766646

[21] Peluso MJ Deitchman AN Torres L Iyer NS Munter SE Nixon CC. Long-term sars-Cov-2-Specific immune and inflammatory responses in individuals recovering from covid-19 with and without post-acute symptoms. Cell Rep (2021) 36(6):109518–. doi: 10.1016/j.celrep.2021.109518 PMC8342976 34358460

[22] Hojjat Jodaylami M Djaïleb A Ricard P Lavallée É Cellier-Goetghebeur S Parker M-F. Cross-reactivity of antibodies from non-hospitalized covid-19 positive individuals against the native, B.1.351, B.1.617.2 and P.1 sars-Cov-2 spike proteins. Sci Rep (2021) 11(1):21601. doi: 10.1038/s41598-021-00844-z 34750399 PMC8575961

[23] Laurie MT Liu J Sunshine S Peng J Black D Mitchell AM. Sars-Cov-2 variant exposures elicit antibody responses with differential cross-neutralization of established and emerging strains including delta and omicron. J Infect Dis (2022) 225:jiab635. doi: 10.1093/infdis/jiab635 PMC8755395 34979030

[24] Faustini S Shields A Banham G Wall N Al-Taei S Tanner C. Cross reactivity of spike glycoprotein induced antibody against delta and omicron variants before and after third sars-Cov-2 vaccine dose in healthy and immunocompromised individuals. J Infect (2022) 84(4):579–613. doi: 10.1016/j.jinf.2022.01.002 PMC8743815 35016901

[25] Zhou Y Han T Chen J Hou C Hua L He S. Clinical and autoimmune characteristics of severe and critical cases of covid-19. Clin Trans Sci (2020) 13(6):1077–86. doi: 10.1111/cts.12805 PMC7264560 32315487

[26] Vlachoyiannopoulos PG Magira E Alexopoulos H Jahaj E Theophilopoulou K Kotanidou A. Autoantibodies related to systemic autoimmune rheumatic diseases in severely ill patients with covid-19. Ann Rheum Dis (2020) 79(12):1661. doi: 10.1136/annrheumdis-2020-218009 32581086

[27] Bastard P Rosen LB Zhang Q Michailidis E Hoffmann H-H Zhang Y. Autoantibodies against type I ifns in patients with life-threatening covid-19. Science (2020) 370(6515):eabd4585. doi: 10.1126/science.abd4585 32972996 PMC7857397

[28] Wang EY Mao T Klein J Dai Y Huck JD Jaycox JR. Diverse functional autoantibodies in patients with covid-19. Nature (2021) 595(7866):283–8. doi: 10.1038/s41586-021-03631-y PMC13130511 34010947

[29] Liu Y Ebinger JE Mostafa R Budde P Gajewski J Walker B. Paradoxical sex-specific patterns of autoantibody response to sars-Cov-2 infection. J Trans Med (2021) 19(1):524. doi: 10.1186/s12967-021-03184-8 PMC8716184 34965855

[30] Chang SE Feng A Meng W Apostolidis SA Mack E Artandi M. New-onset igg autoantibodies in hospitalized patients with covid-19. Nat Commun (2021) 12(1):5417. doi: 10.1038/s41467-021-25509-3 34521836 PMC8440763

[31] Johnston F Anderson T Harlock M Castree N Parry L Marfori T. Lessons learnt from the first Large outbreak of covid-19 in health-care settings in Tasmania, Australia. Western Pac Surveill Response J (2021) 12(4):1–7. doi: 10.5365/wpsar.2021.12.4.884 PMC8873910 35251738

[32] Dong E Du H LF. An interactive web-based dashboard to track covid-19 in real time. Lancet Inf Dis (2020) 20(5):533–4. doi: 10.1016/S1473-3099(20)30120-1 PMC7159018 32087114

[33] Mahase E. Covid-19: What do we know about omicron sublineages? BMJ (2022) 376:o358. doi: 10.1136/bmj.o358 35149516

[34] Smatti MK Cyprian FS Nasrallah GK Al Thani AA Almishal RO Yassine HM. Viruses and autoimmunity: A review on the potential interaction and molecular mechanisms. Viruses (2019) 11(8):762. doi: 10.3390/v11080762 PMC6723519 31430946

[35] Gaebler C Wang Z Lorenzi JCC Muecksch F Finkin S Tokuyama M. Evolution of antibody immunity to sars-Cov-2. Nature (2021) 591(7851):639–44. doi: 10.1038/s41586-021-03207-w PMC8221082 33461210

[36] Siracusano G Brombin C Pastori C Cugnata F Noviello M Tassi E. Profiling antibody response patterns in covid-19: Spike S1-reactive iga signature in the evolution of sars-Cov-2 infection.Front Immunol (2021) 12:772239. doi: 10.3389/fimmu.2021.772239 34804064 PMC8595940

[37] Zhang S Xu K Li C Zhou L Kong X Peng J. Long-term kinetics of sars-Cov-2 antibodies and impact of inactivated vaccine on sars-Cov-2 antibodies based on a covid-19 patients cohort. Front Immunol (2022) 13:829665. doi: 10.3389/fimmu.2022.829665 35154152 PMC8828498

[38] WHO. Who coronavirus disease (Covid-19) dashboard: World health organisation (2020). Available at: https://covid19.who.int/.

[39] Garcia-Beltran WF St. Denis KJ Hoelzemer A Lam EC Nitido AD Sheehan ML. Mrna-based covid-19 vaccine boosters induce neutralizing immunity against sars-Cov-2 omicron variant. Cell (2022) 185(3):457–66.e4. doi: 10.1016/j.cell.2021.12.033 34995482 PMC8733787

[40] Slight-Webb S Lu R Ritterhouse LL Munroe ME Maecker HT Fathman CG. Autoantibody-positive healthy individuals display unique immune profiles that may regulate autoimmunity. Arthritis Rheumatol (2016) 68(10):2492–502. doi: 10.1002/art.39706 PMC5042816 27059145

[41] Neiman M Hellström C Just D Mattsson C Fagerberg L Schuppe-Koistinen I. Individual and stable autoantibody repertoires in healthy individuals. Autoimmunity (2019) 52(1):1–11. doi: 10.1080/08916934.2019.1581774 30835561

[42] Shome M Chung Y Chavan R Park JG Qiu J LaBaer J. Serum autoantibodyome reveals that healthy individuals share common autoantibodies. Cell Rep (2022) 39(9):110873. doi: 10.1016/j.celrep.2022.110873 35649350 PMC9221390

[43] Jukic A Bakiri L Wagner EF Tilg H Adolph TE. Calprotectin: From biomarker to biological function. Gut (2021) 70(10):1978. doi: 10.1136/gutjnl-2021-324855 34145045 PMC8458070

[44] Konikoff MR Denson LA. Role of fecal calprotectin as a biomarker of intestinal inflammation in inflammatory bowel disease. Inflammatory Bowel Dis (2006) 12(6):524–34. doi: 10.1097/00054725-200606000-00013 16775498

[45] Jarlborg M Courvoisier DS Lamacchia C Martinez Prat L Mahler M Bentow C. Serum calprotectin: A promising biomarker in rheumatoid arthritis and axial spondyloarthritis. Arthritis Res Ther (2020) 22(1):105. doi: 10.1186/s13075-020-02190-3 32375861 PMC7201559

[46] Stascheit F Hotter B Hoffmann S Kohler S Lehnerer S Sputtek A. Calprotectin as potential novel biomarker in myasthenia gravis. J Transl Autoimmun (2021) 4:100111–. doi: 10.1016/j.jtauto.2021.100111 PMC8379505 34458711

[47] Shokri-Afra H Alikhani A Moradipoodeh B Noorbakhsh F Fakheri H Moradi-Sardareh H. Elevated fecal and serum calprotectin in covid-19 are not consistent with gastrointestinal symptoms. Sci Rep (2021) 11(1):22001. doi: 10.1038/s41598-021-01231-4 34753964 PMC8578669

[48] Mahler M Meroni P-L Infantino M Buhler KA Fritzler MJ. Circulating calprotectin as a biomarker of covid-19 severity. Expert Rev Clin Immunol (2021) 17(5):431–43. doi: 10.1080/1744666X.2021.1905526 PMC8054493 33750254

