# Supplementary material for: Antibodies against Spike protein correlate with broad autoantigen recognition 8 months post SARS-CoV-2 exposure, and anti-calprotectin autoantibodies associated with better clinical outcomes
Source: Front Immunol. 2022 Aug 11;13:945021. doi: 10.3389/fimmu.2022.945021 (PMC9403331; doi:10.3389/fimmu.2022.945021)
Supplement: Supplementary file 1 [file DataSheet_1.pdf]

Supplementary Material

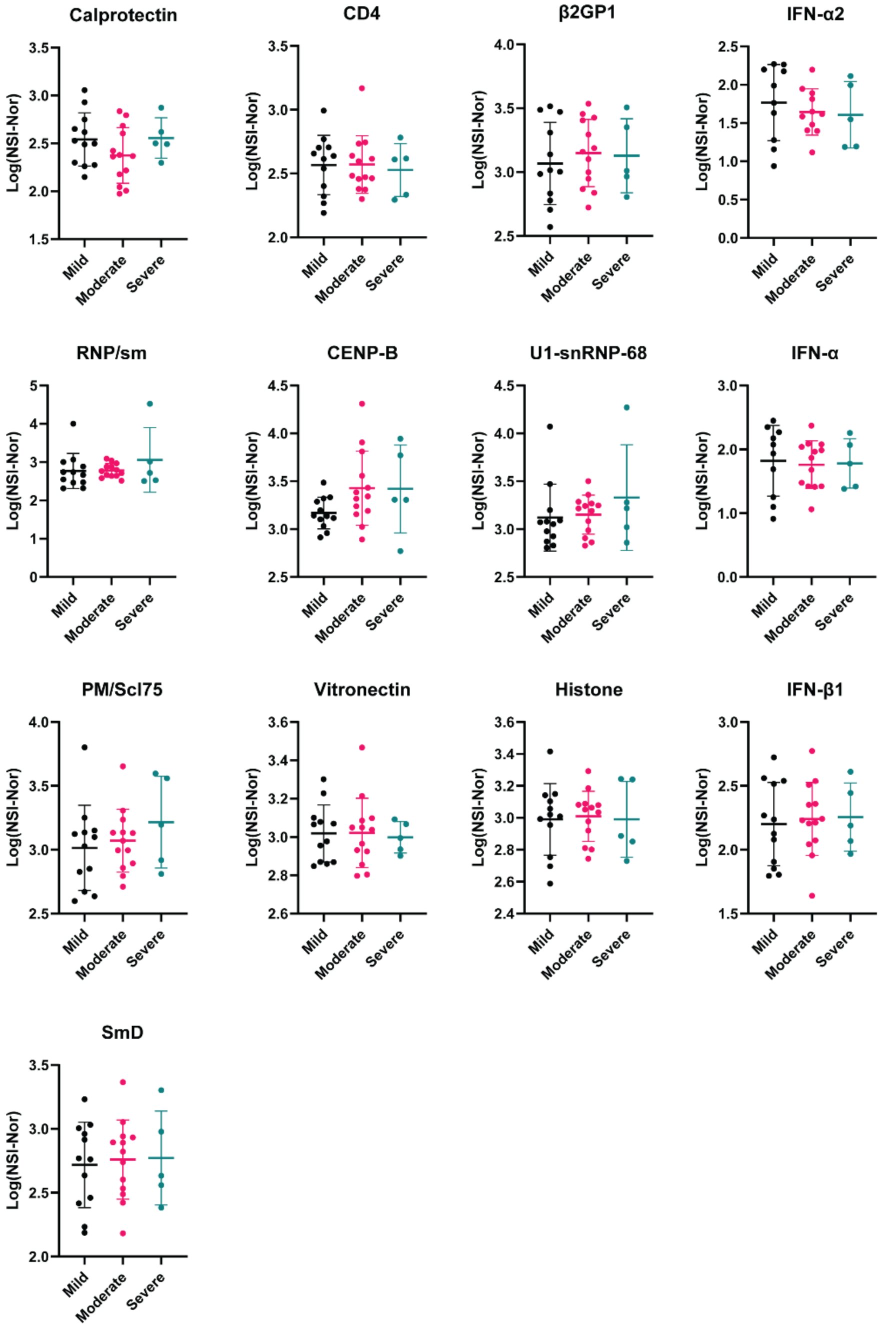

Supplementary Figure 1. Autoantibodies to antigens in COVID-19 convalescent individuals based on symptom severity. Plots of the thirteen top autoantigens comparing reported symptom severity during initial outbreak period (mild, n= 12 ; moderate, n= 13 ; severe, n= 5). Data shown as log transformed NSI-nor values with mean ± standard deviation. Following transformation, normality tested and significance assessed with Kruskal-Wallis test.

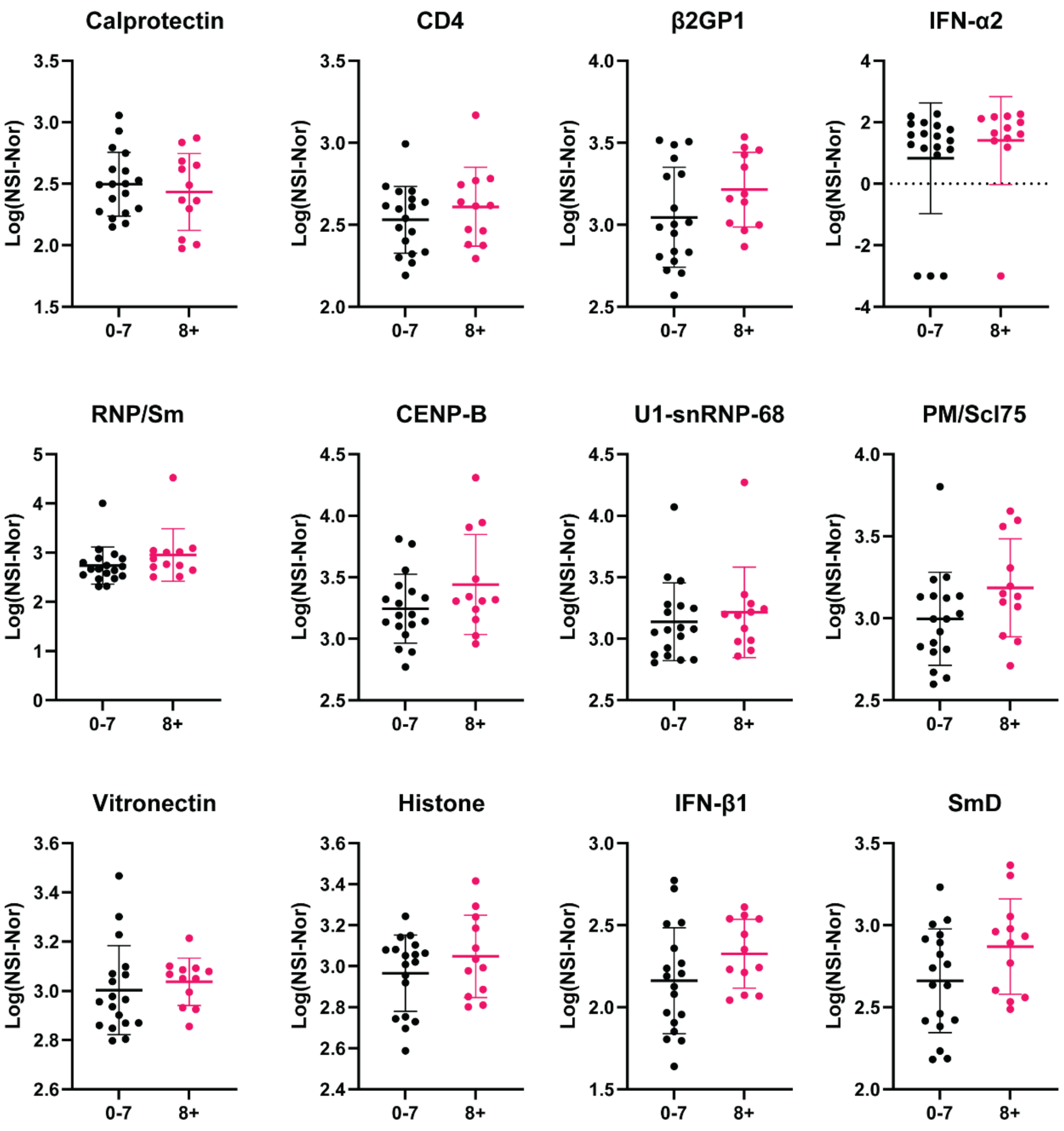

Supplementary Figure 2. Autoantibodies to antigens in COVID-19 convalescent individuals according to number of symptoms experienced. Autoantibody responses to the top autoantigens based on the number of symptoms experienced during initial outbreak period; 0-7, n= 18 and 8+, n= 12. Data shown as log transformed NSI-nor values with mean ± standard deviation. Following transformation, normality tested and significance assessed with either unpaired T test or Mann Whitney test.

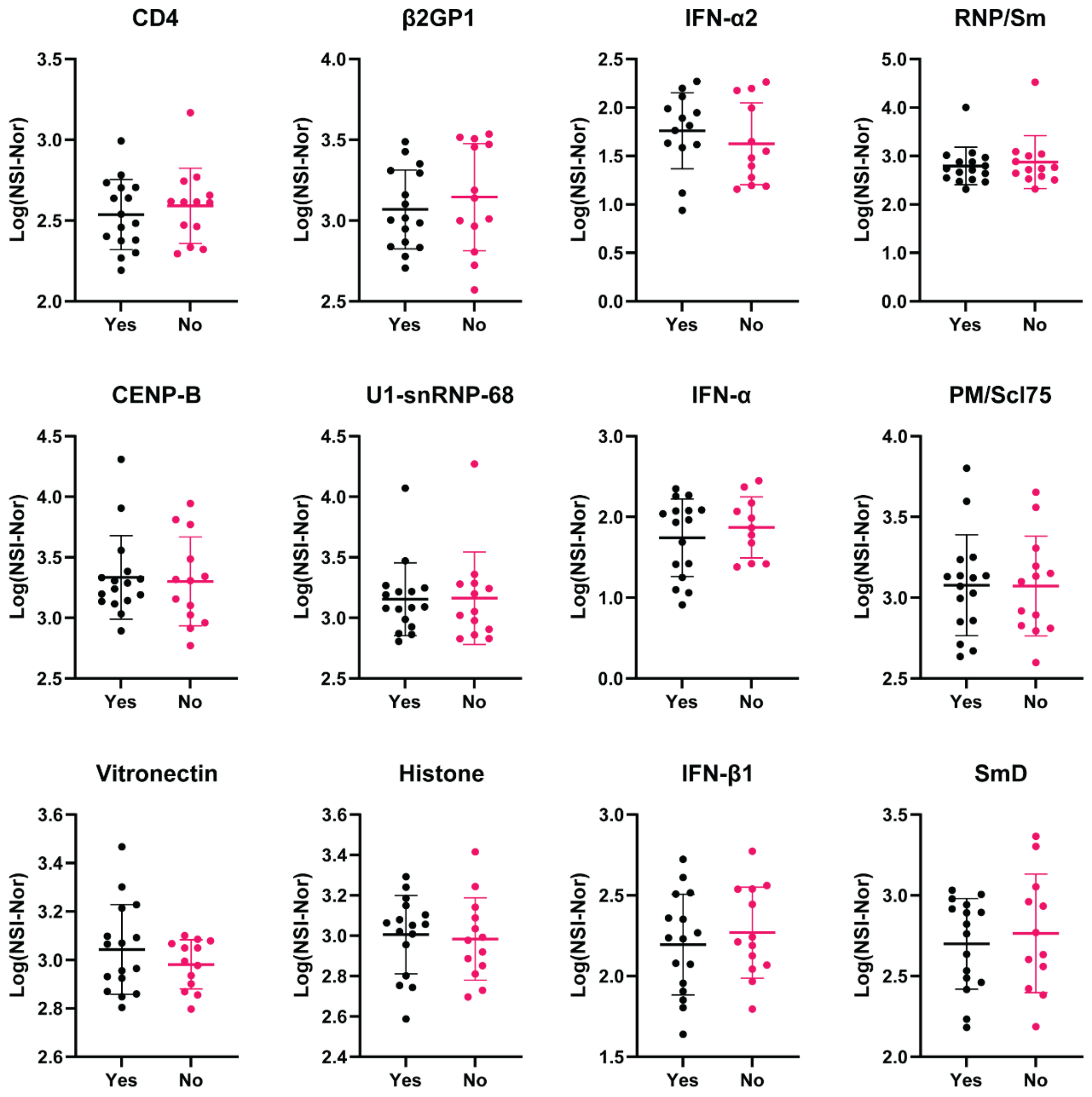

Supplementary Figure 3 . Comparison of autoantibodies to top autoantigens between individuals reported back to normal or not, post COVID-19. Plots of the most common autoantigens, in the present cohort, comparing self-reporting of “Yes” (n= 16) or “No” (n=13) of feeling back to normal post-infection. Data shown as log transformed NSI-nor values with mean ± standard deviation. Following transformation, normality tested and significance assessed with either unpaired T test or Mann-Whitney test.

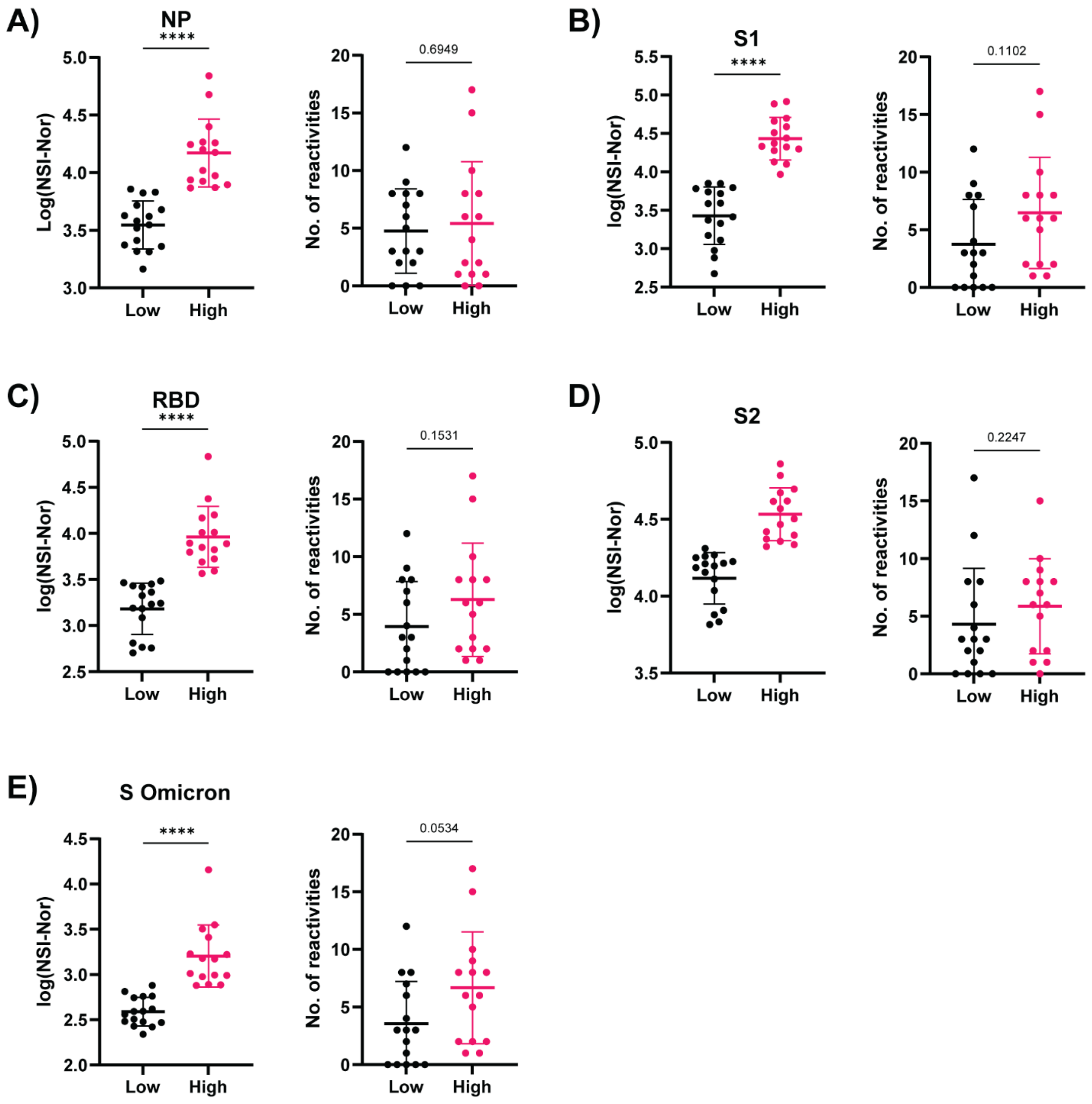

Supplementary Figure 4. Association between level of anti-SARS-CoV-2 antibody responses and the number of positive autoantibody reactivities. COVID-19 convalescent (n=31) divided into high and low anti-Nucleoprotein (NP) (A) and anti-spike subunits, -S1 (B), -RBD (C), -S2 (D) and -S Omicron (E), using the median of responses as the dividing point. The number of autoantibody reactivities within the individuals split into the corresponding high or low group. Normality tested using the Anderson-Darling test prior assessing significance using either unpaired T-test or Mann-Whitney, for normal and non-normal distributed data, respectively. **** p< 0.0001.
